# Comparable Evaluation of Nutritional Benefits of *Lactobacillus plantarum* and *Bacillus toyonensis* Probiotic Supplementation on Growth, Feed Utilization, Health, and Fecal Microbiota in Pre-Weaning Male Calves

**DOI:** 10.3390/ani13213422

**Published:** 2023-11-04

**Authors:** Mohamed S. Ayyat, Hamdy A. El-Nagar, Wael M. Wafa, Khaled M. Abd El-Latif, Samir Mahgoub, Adham A. Al-Sagheer

**Affiliations:** 1 Department of Animal Production, Faculty of Agriculture, Zagazig University, Zagazig 44511, Egypt; 2 Animal Production Research Institute, Agricultural Research Center, Giza 12619, Egypt; 3 Specialized Hospital, Ain Shams University, Cairo 11588, Egypt; 4 Department of Agricultural Microbiology, Faculty of Agriculture, Zagazig University, Zagazig 44511, Egypt

**Keywords:** dairy calf, probiotic, *Lactobacillus plantarum*, Bacillus toyonensis, growth, hematology, immunity, fecal bacteria

## Abstract

**Simple Summary:**

Pre-weaning nutrition plays a crucial role in the growth of male calves, as it shapes their future productivity and overall health. The administration of probiotics to calves during this period can help in maintaining a healthy gastrointestinal tract, establishing a strong immune system, enhancing nutrient absorption, and ultimately leading to optimal growth rates and a successful transition to a productive adult life. This study aims to investigate and compare the blood biochemical, hematological, and fecal microbiota of pre-weaned Holstein male calves supplemented with a mixture of *Lactobacillus plantarum* and *Bacillus toyonensis* versus each individual strain alone. The current study found that using the probiotic mixture displayed superior positive effects compared to each individual strain alone on growth, feed intake, feed efficiency, blood constituents, and modulation of fecal microbiota composition. This approach represents a novel and potentially more effective strategy for enhancing the growth and health of pre-weaned Holstein male calves.

**Abstract:**

This study was conducted to investigate the impact of probiotic supplementation using *Lactobacillus plantarum* DSA 20174 and/or *Bacillus toyonensis* ATCC 55050 on growth performance, blood parameters, hematological measures, and fecal microbiota in pre-weaning Holstein calves. Thirty-two four-day-old male calves with a similar genetic background, weighing an average of 38.27 ± 0.12 kg, were randomly assigned to four groups. The groups consisted of a control group (CON) without supplementation, a group receiving *B. toyonensis* (BT) at 3 × 10^9^ cfu/calf/day, a group receiving *L. plantarum* (LP) at 1 × 10^10^ cfu/calf/day, and a group receiving a combination of LP and BT (LP + BT) at half the dosage for each. The study found that calves supplemented with LP and LP + BT experienced significant improvements in average daily gain and final body weight compared to the control group. The LP + BT group showed the most positive effects on TDMI, starter intake, and CP intake. RBC counts tended to be higher in the probiotic groups, with the LP + BT group having the highest values. The LP + BT group also had higher total protein, albumin, globulin, and hematocrit concentrations. All probiotic groups showed higher serum IgG concentrations. Probiotic supplementation led to increased total bacterial count and decreased levels of *E. coli*, salmonella, and clostridium. The LP + BT group had a significant decrease in coliform count, while both LP and LP + BT groups had increased *Lactobacillus* populations. In conclusion, LP + BT probiotic supplement showed the most beneficial effects on growth, feed efficiency, blood constituents, and modulation of fecal microbiota composition.

## 1. Introduction

Pre-weaned calves have a higher risk of morbidity and mortality during their first few days of life [1,2,3]. Neonatal calves have immature immune and antioxidant systems and low disease resistance. Consequently, these calves are susceptible to respiratory and intestinal ailments, which compromises their subsequent growth and overall health [4]. Antibiotics are used to reduce diarrhea-related morbidity and mortality, but stricter regulations in the livestock sector have led to microbial resistance risks. The EU banned antibiotics and ionophores, while the USA introduced the Veterinary Feed Directive [5]. Therefore, it is essential to offer scientific techniques, like probiotics and natural feed additives, to guarantee optimal growth, development, and health in the first few years of life, as they could have a favorable effect on how the livestock business will evolve in the future [6].

Increased scientific interest in probiotics has persisted for decades due to their potential health advantages. There exist probiotics that are specifically formulated for ruminant animals, which encompass yeast strains such as *Y. lipolytica* and *S. cerevisiae*, as well as bacterial genera including *Bacillus*, *Propionibacterium*, *Bifidobacterium*, *Lactobacillus*, *Enterococcus*, and other noteworthy species including *Megasphaera elsdenii* [7,8]. *Lactobacillus plantarum* has been identified in various silages and fermented foods [9,10]. *L. plantarum* is well known for its ability to endure the process of gastric transit, enabling it to colonize the intestines and positively affect the host [11]. *L. plantarum* has demonstrated beneficial effects in promoting nutrient digestibility, boosting immunological function and mitigating pathogenic colonization across several animal species [12,13]. Moreover, its robustness, resistance to bile and acid, along with the ability to generate antimicrobial compounds [14] render it a highly suitable contender for probiotic supplementation in pre-weaning calves.

*Bacillus* species, including *Bacillus toyonensis*, exhibit potential as probiotics due to their capacity to produce endospores, which allows them to endure adverse environmental circumstances and maintain viability throughout storage and transit within the gastrointestinal system [15]. *Bacillus toyonensis* is a strain of *Bacillus cereus* group that occurs naturally and is characterized by its non-toxigenic and non-pathogenic properties. It has been recognized as a safe additive since it has been found to have no detrimental effects on many animal species [16]. *B. toyonensis* has exhibited probiotic characteristics, including the synthesis of antimicrobial compounds, activation of the immune response, and enhancement of gut health [17,18]. The aforementioned attributes make it a potential additive for augmenting the overall health and performance of pre-weaned calves.

Both investigated probiotics exert their mode of action within the intestines by establishing an unfavorable environment for the pathogenic colonization while simultaneously facilitating the growth of beneficial commensal bacteria. In response, organic acids are produced, which elevate the pH level and stimulates the synthesis of antimicrobial compounds that hinders the growth of pathogenic bacteria. Moreover, probiotics promote the proliferation of favorable bacteria, thus altering the equilibrium of microorganisms within the gut. In addition, they bolster immune function and stimulate immune cells, supporting protection against pathogen-induced or stress-induced intestinal diseases [7,19,20].

Studies have shown that probiotics containing multiple bacterial species and strains are superior to those containing only one strain or species in improving calf health and performance [21,22,23]. However, there has been a lack of research examining the combined effect of supplementary *L. plantarum* and *B. toyonensis* on the performance of pre-weaned dairy calves. Therefore, we hypothesized that supplementing pre-weaning calves with *L. plantarum* and/or *B. toyonensis* probiotics would have a beneficial effect on overall health and performance. The objective of this study was to examine the combination impact of *L. plantarum* and *B. toyonensis*, compared with their individual impacts, on pre-weaning calf growth performance, blood biochemical, hematological, and fecal microbiota.

## 2. Materials and Methods

### 2.1. Probiotic Bacterial and Inoculum Preparation

The *L. plantarum* DSA 20174 and *B. toyonensis* ATCC 55050 strains were acquired from The Center of Bacterial Collection (MERCIN) located at Ain Shams University in Cairo, Egypt. *L. plantarum* was cultivated overnight at a temperature of 37 °C in de Man Rogosa Sharpe broth, as described by De Man et al. [24]. Prior to its use, *L. plantarum* was subcultured three times. *B. toyonensis* was cultivated for a duration of 48 h at a temperature of 37 °C in nutrient broth (NB), which consists of the following components per liter: 5.0 g peptone, 1.0 g beef extract, 2.0 g yeast extract, 5.0 g sodium chloride, with a final pH of 6.8 ± 0.2. The stock cultures of *L. plantarum and B. toyonensis* were kept at −20 °C in 20% glycerol and −80 °C in 10% skim milk with cryoprotectant, respectively. To enumerate *B. toyonensis* and *L. plantarum*, diluted samples were plated on nutrient agar and MRS agar, respectively. The plates were subjected to incubation at a temperature of 37 °C for 48 h. The cell density, as determined through plate counting, was ~10^8^ CFU/mL. This inoculum ratio was used in all experiments of dosing animals.

### 2.2. Experimental Design, Animals, and Treatment Diets

The current study was conducted at the El-Gemmiza Research Station located in El-Santa, Gharbia Governorate, Egypt, which is a part of the Agriculture Research Center’s Animal Production Research Institute in Giza. The research protocol that involved animals was in accordance with the experimental recommendations of the Animal Ethics Committee of Zagazig University (ZU-IACUC). This study was performed using a completely randomized design involving 32 male Holstein calves with a similar genetic background, approximately four days old. The calves were allocated into four different treatment groups, each consisting of eight calves. The overall mean weight of the calves was 38.27 ± 0.12 kg at the start of the study. Immediately after birth, the calves were given colostrum amounting to 10% of their birth body weight, followed by transition milk until the third day of life. On the third day, they were transported to the experimental facility where they were housed for a duration of 84 days.

The experimental groups consisted of a control group (CON) without supplementation, a group receiving *B. toyonensis* (BT) at 3 × 10^9^ cfu/calf/day, a group receiving *L. plantarum* (LP) at 1 × 10^10^ cfu/calf/day, and a group receiving a combination of LP and BT (LP + BT) at half the dosage for each. The tested probiotics dosage was selected based on the findings of Jiang, et al. and Novak, et al. [25,26]. During the period of 4 to 60 days of age, the milk was supplemented with the tested probiotics before morning feeding. Subsequently, from 61 to 88 days of age, the probiotics were added to the starter mixture that was fed to calves every morning.

Calves were raised in individual pens (1.5 m length × 1.0 m width) with rice straw bedding for the duration of the trial. Fresh water was made available to the calves at all times through buckets. Throughout the experiment, the calves were provided with starter mixture ad libitum, which was formulated based on the recommendations of NRC [27]. Table 1 presents the ingredients and chemical composition the starter mixture.

The chemical composition of the starter mixture was estimated by AOAC [28] procedures using representative samples that were milled to pass a 1 mm screen. The starter samples were analyzed for crude ash (method no. 954.01), DM contents (method no. 925.10), ether extract content (method no. 920.39) using petroleum ether in a Soxhlet apparatus, and crude protein (N × 6.25) using the Kjeldahl procedure (method no. 954.01). The quantification of neutral detergent fiber (NDF) and acid detergent fiber (ADF) was done using the procedures described by Van Soest, et al. and the AOAC [28,29] method 973.18), respectively. All the chemical analyses were performed on DM basis.

### 2.3. Growth Performance and Feed Intake 

The calves’ weights were recorded before morning feedings on d 4 of age (initial weight) and thereafter every 28 days (d 32, d 60, and d 88 of age). Data of starter mixture intake were collected for each calf every four weeks. The difference between the amount of starter mixture offered and the orts was calculated and recorded as an average on a grams-per-day basis for each calf. The total dry matter intake (DMI) included both starter mixture and milk. The calculation of the average daily gain (ADG) involved dividing the difference between the final and initial body weight by the duration in days. The calculation of feed efficiency (FE) was done by dividing ADG in kg by the amount of TDMI in kg. These measurements were taken for four different time periods: d 4–32, d 33–60, d 61–88, and d 4–88 (overall period).

### 2.4. Hematological Study and Biochemical Assay

Blood specimens were drawn 3 h ± 30 min after morning feeding from the jugular vein of 20 calves (*n* = 5 calves per group) at 32, 60, and 88 days of age. Blood was drawn into tubes with K3-EDTA for hematological profile or without anticoagulant (for serum collection to measure biochemical and immunological parameters). Samples without anticoagulant were centrifuged at 3000× *g* at 4 °C for 15 min. The resulting supernatant was carefully collected and kept in a deep freezer at −20 °C for subsequent serum measurements. The hematological profiles were assessed using an automated hematology analyzer (Medonic CA620 VET, Stockholm, Sweden). The following parameters were measured: red blood cell (RBC) count, hemoglobin (Hb) level, hematocrit (Hct) value, mean corpuscular hemoglobin concentration (MCHC), platelet count, mean corpuscular hemoglobin (MCH), mean corpuscular volume (MCV), white blood cell (WBC) count, and proportion of basophils, neutrophils, lymphocytes, monocytes, and eosinophils.

The contents of total cholesterol (TC), triglyceride (TG), total protein, albumin, urea, high-density lipoprotein (HDL), blood urea nitrogen (BUN), very low-density lipoprotein (VLDL), and low-density lipoprotein (LDL) were estimated from the sera samples using commercial kits (Diamond Diagnostic, Dokki, Giza, Egypt) following the manufacturer’s instructions. To determine the globulin levels, the total protein levels were subtracted from the serum albumin levels. Additionally, the serum concentration of immunoglobulin G (IgG) was determined using commercial ELISA kits according to the manufacturer’s directives. 

### 2.5. Enumeration of Probiotic and Associated Bacteria in Calf Feces and Cloacal Swabs

For detection of total bacterial count (TBC), coliform count (CFC), Lactobacilli count (LC), spore-forming bacteria (SFB: *Bacillus* and *Clostridium*), *Salmonella,* and *Escherichia coli* in animal samples, the calf feces, and cloacal swabs were mixed into buffered peptone water (8.5 g sodium chloride/L and 1.0 g peptone/L) and incubated at 35–37 °C for 18–24 h. There are two steps for detecting the tested bacteria. The first step: before incubation, total cultivable bacteria TBC, CFC, Lactobacilli, and spore-forming bacteria count (SFB) *Escherichia coli* and *Salmonella* were counted. TBC was counted onto plate count agar (Merck, 1.05463, Darmstadt, Germany) for 48 h at 30 °C. *Bacillus* spp. count was determined after pasteurizing the samples at 80 °C for 15 min onto plate count agar (Merck, 1.05463, Darmstadt, Germany) for 48 h at 30 °C. Coliform counts were taken following incubation on Violet Red Bile Agar (VRB, Biolife, Italy) for 24 h at 37 °C. After 24 h of incubation at 37 °C, the number of *E. coli* colonies on eosin-methylene blue agar plates (Merck, Darmstadt, Germany) was recorded. After 24 h of incubation at 37 °C, *Salmonella* spp. was counted on Xylose Lysine Deoxycholate agar (XLD: Biolife, Italy). *Clostridium* was counted onto CHROMagar™, following 24 h of anaerobic incubation at 35–37 °C, the colonies could be read under UV light. Excel 2010 was used to convert the bacterial count to a Log_10_ before performing statistical analysis. The second step: after incubation, the samples at 37 °C for 18–24 h, 100 μL were streaked onto eosin-methylene blue agar plates, Xylose Lysine Deoxycholate agar plates and CHROMagar™ for *E. coli*, *Salmonella* spp. and *Clostridium*, respectively. Common colony types and morphological features were analyzed in the plates. *E. coli* and *Salmonella* colonies were identified using citrate, Voges–Proskauer, methyl red, and indole reaction biochemical assays, and their identities were confirmed using the standards set out by ISO 16654 and ISO 6579, respectively [30,31].

### 2.6. Statistical Analysis

The data of growth performance, starter intake, DMI, FE, hematological parameters, and serum measures were subjected to analysis using the general linear model method of SPSS statistical software (version, 20) by considering the period as the repeated measure. The repeated measures model contained fixed effects of treatment, period, and interaction of period× treatment, and the calf identity was considered as a random effect. Each individual calf served as an independent experimental unit. The following model was adopted:

Y_ijkl_ = µ + Calf_i_ + P_j_ + T_k_ + (P × T)_jk_ + e_ijkl_ (1)

where Y_ijkl_ is the observation; µ is the overall mean; P_j_ is the effect of period; T_k_ is the effect of treatment; (P × T)_jk_ is the effect of interaction between treatment and period; e_ijkl_ is the error term. The data concerning fecal microbial populations were analyzed using the same model, excluding the effect of the experimental period as follows:

Y_ijkl_ = µ + Calf_i_ + T_k_ + e_ijkl_ (2)

The data are reported as the least squares means along with their standard error of the mean. The comparison of the means of treatment groups was conducted using Tukey’s multiple range test, and statistical differences were evaluated at a significance level of *p* < 0.05. Statistical trends were identified in the range of *p*-values from 0.05 to 0.10.

## 3. Results

### 3.1. Growth Performance and Feed Intake 

**Figure 1:**
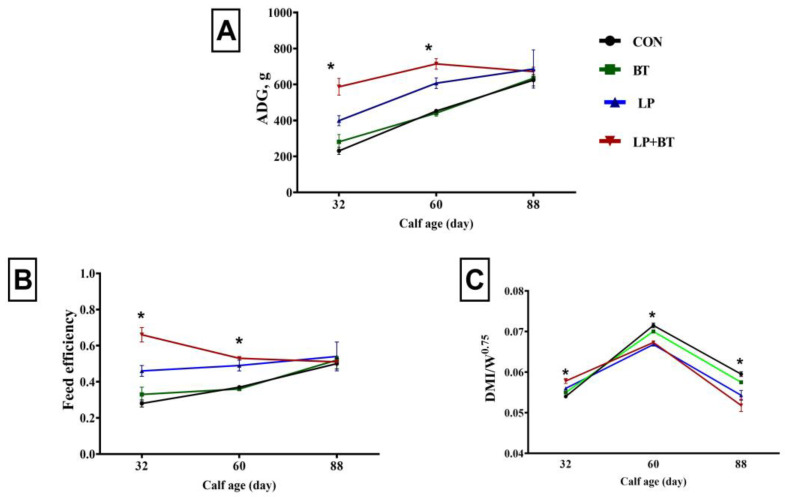
Changes in average daily gain (ADG, **A**), feed efficiency (**B**), and dry matter intake per kilogram of BW^0.75^ (**C**) in pre-weaned calves supplemented without (CON group) or with *Bacillus toyonensis* (BT) at a dosage of 3 × 10^9^ cfu/calf/day, *Lactobacillus plantarum* (LP) at a dosage of 1 × 10^10^ cfu/calf/day, and their combination (LP + BT) at half the dosage for each from day 4 to day 88 of age. Error bars indicate the SEM; *, *p* < 0.05.

As presented in Table 2, calves supplemented with LP and LP+ BT exhibited a significant increase in both ADG and final weight compared to the control group (*p* < 0.01). Throughout the experimental period, all calves received an equal daily allowance of milk, while the starter mixture was made available to them ad libitum. However, the treatments had a significant impact on TDMI, starter intake, and CP intake (*p* < 0.01). Notably, the calves supplemented with the probiotic mixture (LP + BT group) displayed the most prominent positive effects when compared to the control group. The individual addition of LP and BT did not affect the TDMI, the starter intake, or the CP intake. There was a significant reduction in the values of TDMI/W^0.75^ in the LP and LP+ BT groups compared to the other groups (*p* < 0.01). The LP+ BT group showed a higher FE compared to the other groups (*p* < 0.01; Table 2). Compared to the other groups, the LP and LP+ BT treatment groups showed a significant increase in ADG (*p* = 0.012) and FE (*p* < 0.01) during the periods from d 4 to d 32 and 33 d to 60 d (Figure 1A,B). From d 4 to d 32, the TDMI/W^0.75^ values of LP + BT calves were higher, but from d 32 to d 60 and d 61 to d 88, all probiotic groups had lower TDMI/W^0.75^ values (*p* < 0.01) compared to the CON group (Figure 1C).

### 3.2. Hematological Parameters

The effects of LP and/or BT supplementation on the hematological components of pre-weaned calves throughout the experimental period are shown in Table 3. There was no effect of treatment on most hematological parameters including Hb, MCV, MCH, MCHC, platelet, WBCs, lymphocyte, neutrophils monocytes, eosinophils, and basophils (*p* > 0.05). However, a trend (*p* = 0.072) towards an increase in RBC counts was observed in the probiotic-supplemented groups, with the highest values recorded in the mixture group (LP+ BT). The calves in the LP+ BT group exhibited significantly elevated Hct concentrations (*p* = 0.028) compared to the calves in the CON group.

### 3.3. Blood Biochemical Parameters

**Figure 2:**
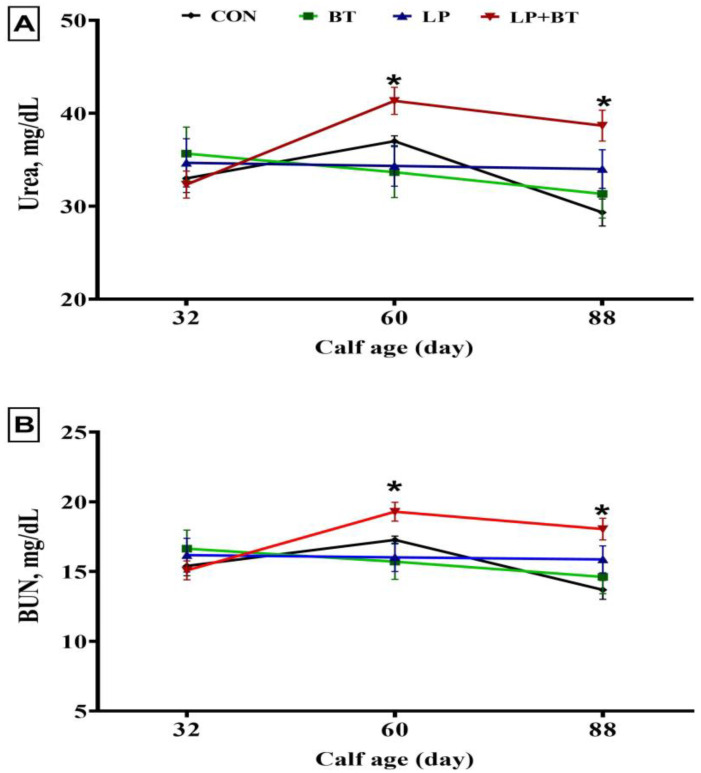
Changes in concentrations of blood urea (**A**) and urea nitrogen (BUN, (**B**)) in pre-weaned calves supplemented without (CON group) or with *Bacillus toyonensis* (BT) at a dosage of 3 × 10^9^ cfu/calf/day, *Lactobacillus plantarum* (LP) at a dosage of 1 × 10^10^ cfu/calf/day, and their combination (LP + BT) at half the dosage for each from day 4 to day 88 of age. Error bars indicate the SEM; *, *p* = 0.066.

As shown in Table 4, serum total protein, albumin, and globulin values significantly increased in the group receiving LP + BT (*p* < 0.05). All treatment groups that received probiotic supplementation experienced a significant elevation in serum concentration of IgG in comparison to the CON group (*p* = 0.013). The treatment did not have a significant effect (*p* > 0.05) on serum concentrations of, TC, TG, HDL, LDL, VLDL, and albumin to globulin ratio (Table 4). There was a tendency for higher serum concentrations of urea and BUN in the LP + BT group compared to the other group on days 60 and 88 (*p* = 0.067; Figure 2).

### 3.4. Fecal Bacterial Microbiota

Table 5 summarizes the results of fecal microbial population in pre-weaned calves supplemented with LP and/or BT. Significant increases in TBC (*p* < 0.001) and decreases in the count of *E. coli* (*p* < 0.001), Salmonella (*p* < 0.001), and Clostridium (*p* = 0.005) were observed in all probiotic groups. The LP + BT group had a significant decrease in coliform count compared to the other groups (*p* = 0.005). The count of *Lactobacillus* populations was significantly increased in both the LP and LP + BT groups when compared to the other groups (*p* < 0.001). Additionally, the BT group had a significantly higher fecal *bacillus* count, while the LP + BT group had a lower count compared to the CON group (*p* = 0.001).

## 4. Discussion

In the present work, calves received milk or starter supplemented with LP and LP + BT displayed a significantly enhanced ADG and final weight than those fed non-supplemented diets. Likewise, in a study by Casper et al. [32], it was observed that neonatal Holstein calves receiving *L. plantarum* GB LP-1 supplementation at a dosage of 8 g/d exhibited a significant increase of over 14% in ADG compared to the CON group during the 0-to-56-day period of the experiment. According to Cangiano et al. [33], 5 of the 11 studies that investigated the benefits of probiotics demonstrated significant improvements in ADG. Additionally, Timmerman et al. [23] demonstrated that feeding pre-weaning Holstein Friesian calves a combination of five *Lactobacillus* strains and *Enterococcus faecium* resulted in enhanced ADG. LP-enhancing effect on ADG is thought to occur through enhanced gut health by limiting pathogen invasion [33]. It could additionally be attributable to the decrease in intestinal viscosity and the increase in crypt depth and villus height [34]. Previous research using LP has demonstrated substantial nutritional advantages of greater weight gains and energy and protein retention while reducing *Salmonella* and *Clostridia counts* when fed to stressed swine [35]. In accordance with the present study, the analysis of the fecal microbial population in pre-weaned calves fed whole milk or starter fortified with LP, BT, and LP + BT showed decreases in the count of *E. coli*, *Salmonella*, and *Clostridium* with the maximum reduction of coliform count in the LP + BT group. In this regard, LP has been demonstrated to exhibit a wide range of antibacterial activities across several hosts [36,37]. LP has been reported to exert inhibitory effects on the proliferation of the prevalent intestinal pathogen. This is achieved by its antibacterial metabolism, creation of a lower pH condition through hydrogen peroxide production, and its ability to effectively cling to intestinal cells [35]. The growth-promoting activity in LP and LP + BT could be also related the enhanced FE in both groups and increased TDMI, starter intake, and CP intake in the LP + BT. Similarly, a significant enhancement was recorded in the feed conversion in veal calves fed milk replacers fortified with five strains of *Lactobacillus* and *Enterococcus faecium* as a probiotic [23]. In this respect, Cangiano et al. [33] reported that feeding microbial-based probiotics promoted stable gut microbiota in dairy calf, thereby enhancing digestive efficiency. Interestingly, the pronounced growth-enhancing effect in the calves that received the combination of the two probiotic LP and BT could be highly related to the increased TDMI. In this regard, Raza et al. [38] reported that increased TDMI in the pre-weaned calves could potentially stimulate rumen development and subsequently enhance growth performance.

Blood metabolites, including blood total protein, albumin, globulin, urea, and BUN are indicators of fermentative changes and microbial protein synthesis occurring in the developing rumen in pre-weaned calves [39]. The concentration of BUN can serve as a useful tool for assessing the efficacy of dietary protein utilization in young calves [40]. The efficient functioning of the rumen has also been linked to elevated levels of BUN [38]. Increased levels of BUN in the group that received LP + BT indicate improved utilization and metabolism of proteins. Increased BUN levels indicate an enhanced rate of protein catabolism and utilization. The potential impact of probiotics on protein metabolism depends on their ability to enhance the efficient breakdown of dietary protein into amino acids, which can then be utilized for a range of physiological functions such as tissue growth and repair [41]. In the current investigation, it was shown that the pre-weaned calves fed milk or starter supplemented with LP + BT had higher levels of serum total protein, albumin, and BUN than the control calves and higher serum albumin levels of albumin in LP group. The aforementioned findings could be explained by an increased intake of starter and CP, leading to larger hydrolysis of dietary proteins or the conversion of amino acids into ammonia through deamination [42]. Importantly, the other blood indices including TC, TG, HDL, LDL, VLDL, and albumin to globulin ratio as well as the erythrogram and leukogram components exhibited values within the average range, as previously documented in pre-weaning calves [40,43]. This reflects the safety of this probiotic combination. Notably, an increasing trend in RBC counts was observed in the probiotic-supplemented groups, with the highest values recorded in the mixture group (LP + BT). The latter group also exhibited significantly elevated Hct concentrations than the calves in the CON group. The favorable effect of LP+ BT on the gut microbiota, which has a significant influence on the metabolism and absorption of iron, the key component of erythrocytes Hct, could be responsible for the improvement of RBC count and Hct concentration [44]. In line with this, LP treatment has been reported to increase the iron absorption in the body [45].

The neonatal phase of calf growth is a critical stage characterized by a decreased immune response, which can lead to serious health issues such as respiratory and intestinal distress leading to high morbidity and mortality [38]. The results of our study showed that adding LP, BT, or their combination in whole milk or starter improved newborn health by increasing serum IgG and globulin levels compared to the CON group. In previous studies, multispecies probiotic supplementation in pre-weaning dairy calves was associated with an increase in IgA levels [46], which is in line with our results. Also, Hong et al. [47] reported that the supplementation of probiotics in milk fortification was associated with elevated IgG levels, indicating an enhanced immune response against spores.

In this regard, Cangiano et al. [33] proposed that the supplementing of lactic acid bacteria may have a greater effect throughout the lower gastrointestinal tract, as it has the potential to influence systemic immune function. This is achieved by enhancing both humoral and cell-mediated immunity through the promotion of B and T cell activity, as well as the reduction of cortisol levels in the bloodstream. Lactic acid bacteria have been shown to interact with intestinal resident microflora and immunological and epithelial cells, as well as initiate and promote immune function, resulting in antibody formation [48]. Moreover, the positive effects of *Bacillus* probiotic strains in stimulating immunity in calves have been reported [49].

Notably, the pre-weaned calves supplemented with the probiotic mixture (LP + BT group) displayed the most prominent positive effects when compared to the groups fed the individual probiotic. The beneficial impact on calf health may have resulted from the synergistic effects observed when a combination of probiotics is added to milk and then starter mixture, as observed in the present study. This could possibly lead to increased starter consumption, hence potentially inducing favorable alterations in the rumen microbial population. Comparably, Laarman and Oba [50] confirmed that ruminal fermentation parameters and microbial population were enhanced by the increased consumption of milk replacer and starter feed, as in our study, in pre-weaned calves. Another important issue to note is some earlier reports proposed that using a probiotic blend of anaerobic (LP) and aerobic (BT) strains of bacteria may have synergistic beneficial effects on animal health, growth, and welfare [51].

## 5. Conclusions

This study demonstrated that the supplementation of pre-weaning Holstein calves with a probiotic combination of *Lactobacillus plantarum* and *Bacillus toyonensis* had significant positive effects on growth, feed efficiency, blood constituents, and modulation of fecal microbiota composition. These findings support the potential use of LP + BT as a beneficial probiotic strategy to enhance calf performance and health during the critical pre-weaning period. Still, more research with direct assessments of rumen development in calves given a probiotic combination is required.

## Figures and Tables

**Table 1:** Ingredients chemical composition of the starter mixture fed to calves.

Item	Value
Ingredient, g/kg	
Yellow corn	350
Soybean meal, 44% CP	250
Alfalfa hay	150
Wheat bran	95
Barley grain	100
Molasses	30
Iodized sodium chloride	8
Calcium phosphate	10
Mineral and vitamin premix ^1^	7
Total	1000
Chemical analysis, g/kg of air dry basis	
Dry matter	904.8
Organic matter	939.6
Crude protein	190.8
Ether extract	29.9
Ash	60.3
Neutral detergent fiber	248.5
Acid detergent fiber	128.6

^1^ Each kilogram of premix contains: vitamin A, 50,000 IU; vitamin D3, 10,000 IU; vitamin E, 2900 IU; Ca, 196 g; P, 96 g; MgSO_4_, 19,000 mg; FeSO_4_, 9000 mg; CuSO_4_, 5000 mg; Mn, 6000 mg; ZnSO_4_, 8000 mg; Co, 20 mg; I, 227 mg; Se, 67 mg.

**Table 2:** The feed intake and growth performance of non-supplemented calves and calves supplemented with *Bacillus toyonensis* (BT), *Lactobacillus plantarum* (LP), and their combination (LP + BT).

	Treatment (Trt)				SEM	*p*-Value		
	CON	BT	LP	LP + BT		Trt	Period	Trt × Period
Initial BW (kg)	38.20	38.47	38.17	38.23	0.12	0.850	-	-
Final BW (kg)	74.80 ^c^	76.43 ^c^	85.53 ^b^	93.47 ^a^	2.11	<0.01	-	-
ADG; (g/d)	435.7 ^c^	452.0 ^c^	563.9 ^b^	657.5 ^a^	24.91	<0.01	<0.01	0.012
TDMI ^2^ (g/d)	1099.5 ^b^	1101.7 ^b^	1121.8 ^b^	1183.2 ^a^	28.93	<0.01	<0.01	0.183
Starter DMI (g/d)	416.8 ^b^	419.1 ^b^	439.0 ^b^	500.6 ^a^	40.31	<0.01	<0.01	0.184
CP intake (g/d)	244.8 ^b^	245.2 ^b^	249.1 ^b^	260.8 ^a^	5.16	<0.01	<0.01	0.183
DMI/W^0.75^	0.062 ^a^	0.061 ^a^	0.059 ^b^	0.059 ^b^	0.001	<0.01	<0.01	<0.01
Feed efficiency ^3^	0.384 ^c^	0.403 ^c^	0.497 ^b^	0.567 ^a^	0.017	<0.01	<0.01	<0.01

^a–c^ Means within the row with different letters differed significantly (*p* < 0.05). SEM: standard error of the mean; ADG, average daily gain; TDMI, total dry matter intake; Starter DMI, starter dry matter intake; CP intake, crude protein intake. ^2^ TDMI: total dry matter intake of milk replacer and starter feed, g/d. ^3^ Feed efficiency: g of ADG/g of TDMI.

**Table 3:** Changes in hematological parameters of non-supplemented calves (CON) and calves supplemented with *Bacillus toyonensis* (BT), *Lactobacillus plantarum* (LP), and their combination (LP + BT).

	Treatment (Trt)				SEM	*p* Value		
	CON	BT	LP	LP + BT		Trt	Period	Trt × Period
Erythrogram								
RBCs, 10^6^ × µL	4.74	5.18	5.51	5.54	0.13	0.072	0.026	0.677
Hb, g/dL	7.83	8.66	9.03	9.45	0.30	0.120	0.003	0.146
Hct, %	28.19 ^b^	29.26 ^b^	30.02 ^ab^	32.59 ^a^	0.85	0.028	<0.001	0.118
MCV, fL	59.61	56.44	54.59	59.63	1.27	0.105	<0.001	0.343
MCH, pg/dL	16.37	16.70	16.37	17.00	0.28	0.785	0.055	0.130
MCHC, g/dL	27.96	29.81	30.14	29.97	0.70	0.507	<0.001	0.458
Platelet, 10^3^/µL	342.22	321.22	350.44	378.78	21.18	0.643	<0.001	0.131
Leukogram								
WBCs, 10^3^/µL	9.84	9.90	10.89	10.65	0.33	0.657	0.540	0.900
Lymphocyte, %	58.22	55.54	56.83	59.43	0.95	0.529	0.389	0.538
Neutrophils, %	36.64	39.26	37.82	35.21	1.02	0.575	0.354	0.574
Monocytes, %	3.24	3.36	3.49	3.34	0.10	0.862	0.442	0.659
Eosinophils, %	1.71	1.65	1.66	1.83	0.06	0.442	<0.001	0.133
Basophils, %	0.18	0.19	0.20	0.20	0.01	0.774	<0.001	0.193

^a,b^ Means within the row with different letters differed significantly (*p* < 0.05). SEM stands for standard error of the mean; Hb, hemoglobin; RBCs: red blood cells; Hct: the hematocrit; MCV: mean corpuscular volume; MCH: mean corpuscular hemoglobin; MCHC: mean corpuscular hemoglobin concentration; WBCs: white blood cells.

**Table 4:** Changes in selected measures in the serum of non-supplemented calves (CON) and calves supplemented with *Bacillus toyonensis* (BT), *Lactobacillus plantarum* (LP), and their combination (LP + BT).

	Treatment (Trt)				SEM	*p*-Value		
	CON	BT	LP	LP + BT		Trt	Period	Trt × Period
TP, g/dL	5.39 ^b^	5.60 ^b^	5.64 ^b^	6.18 ^a^	0.09	0.003	0.002	0.508
ALB, g/dL	2.94 ^b^	2.93 ^b^	2.92 ^b^	3.27 ^a^	0.05	0.003	0.001	0.411
GLOB, g/dL	2.45 ^b^	2.68 ^ab^	2.72 ^ab^	2.91 ^a^	0.05	0.016	0.046	0.757
A/G	1.21	1.10	1.09	1.12	0.02	0.112	0.339	0.884
TC, mg/dL	81.56	83.11	80.00	79.44	1.11	0.458	0.001	0.185
TG, mg/dL	82.89	87.44	87.67	88.11	1.67	0.700	0.539	0.574
HDL, mg/dL	42.22	39.78	41.22	45.33	1.07	0.188	0.009	0.198
LDL, mg/dL	21.49	23.29	19.96	18.09	1.30	0.515	0.158	0.305
VLDL, mg/dL	17.84	20.04	18.82	16.02	1.20	0.690	0.155	0.563
Urea, mg/dL	33.11	33.56	34.33	37.44	0.72	0.065	0.076	0.067
BUN, mg/dL	15.45	15.66	16.02	17.47	0.33	0.065	0.076	0.066
IgG, mg/mL	21.86 ^b^	29.34 ^a^	30.17 ^a^	31.64 ^a^	1.17	0.013	0.950	0.362

^a,b^ Means within the row with different letters differed significantly (*p* < 0.05). SEM stands for standard error of the mean; TP: total protein; ALB: albumin; GLOB: globulin; A/G: albumin/globulin ratio; TC: total cholesterol; TG: total triglycerides; HDL: high-density lipoprotein; LDL: low-density lipoprotein; VLDL: very low-density lipoprotein; BUN: blood urea nitrogen; IgG: immunoglobulin G.

**Table 5:** Fecal bacterial microbiota (Log cfu/g) of non-supplemented calves (CON) and calves supplemented with *Bacillus toyonensis* (BT), *Lactobacillus plantarum* (LP), and their combination (LP + BT).

	Treatment				SEM	*p*-Value
	CON	BT	LP	LP + BT		
Total bacterial count	8.24 ^d^	10.71 ^b^	9.56 ^c^	10.90 ^a^	0.321	<0.001
Coliforms	5.79 ^a^	5.25 ^a^	5.37 ^a^	4.39 ^b^	0.176	0.008
Lactobacillus	5.69 ^b^	6.31 ^b^	7.89 ^a^	7.94 ^a^	0.308	<0.001
Bacillus	5.66 ^b^	7.87 ^a^	5.92 ^b^	4.62 ^c^	0.379	0.001
*E. coli*	5.87 ^a^	4.26 ^b^	4.11 ^c^	3.12 ^d^	0.298	<0.001
*Salmonella* spp.	5.56 ^a^	3.41 ^b^	3.21 ^b^	3.18 ^b^	0.309	<0.001
Clostridium	5.03 ^a^	3.76 ^b^	3.67 ^b^	3.40 ^b^	0.214	0.005

^a–d^ Means within the row with different letters differed significantly (*p* < 0.05). SEM: standard error of the mean.

## Data Availability

The data that support the findings of this study are available upon reasonable request.
